# Backbone assignments, and effect of Asn deamidation, of the N-terminal region of the partitioning protein IncC1 from the plasmid RK2

**DOI:** 10.1007/s12104-021-10021-y

**Published:** 2021-04-15

**Authors:** M. Fayyaz Rehman, M. Jeeves, E. I. Hyde

**Affiliations:** 1 School of Biosciences, University of Birmingham, Edgbaston, Birmingham, B15 2TT UK; 2 Henry Wellcome NMR Centre, University of Birmingham, Edgbaston, Birmingham, B15 2TT UK; 3 Present Address: Institute of Chemistry, University of Sargodha, Sargodha, Punjab Pakistan

**Keywords:** ParA, IncC, Deamidation, Intrinsic disorder, IsoAspartate

## Abstract

IncC from the low-copy number plasmid RK2, is a member of the ParA family of proteins required for partitioning DNA in many bacteria and plasmids. It is an ATPase that binds DNA and its ParB protein partner, KorB. Together, the proteins move replicated DNA to appropriate cellular positions, so that each daughter cell inherits a copy on cell division. IncC from RK2 is expressed in two forms. IncC2 is homologous to bacterial ParA proteins, while IncC1 has an N-terminal extension of 105 amino acids and is similar in length to ParA homologues in other plasmids. We have been examining the role of this extension, here called IncC NTD. We present its backbone NMR chemical shift assignments and show that it is entirely intrinsically disordered. The assignments were achieved using C-detected, CON-based spectra, complemented by HNN spectra to obtain connectivities from three adjacent amino acids. We also observed evidence of deamidation of the protein at a GNGG sequence, to give isoAsp, giving 2 sets of peaks for residues up to 5 amino acids on either side of the modification. We have assigned resonances from around the position of modification for this form of the protein.

## Biological context

The partitioning of DNA to daughter cells is a vital process for all dividing organisms. In most bacteria this requires an ATPase from the ParA family of proteins, and a DNA-binding protein from the ParB family of proteins that recognises a specific, centromere-like, DNA site and stimulates the activity of the ATPase. The exact mechanism of this process is poorly understood and much has been learnt from the study of the process in low copy number plasmids, such as RK2. Many plasmids encode ParA and ParB protein homologues, in most of which the ParA protein contains an N-terminal extension, not found in bacteria. IncC from RK2 is unusual in that it is expressed in two forms from different start codons within the same gene (Thomas and Smith 1986). Both proteins are found, but in slightly different ratios in different hosts. The shorter IncC2 protein is similar to bacterial ParA proteins while IncC1 contains an additional 105 amino acids, here called IncC NTD. The identical C-terminal sequence of the two proteins makes this a unique system to determine the effect of the N-terminal extension on protein structure and activity. This region of IncC1 is intrinsically disordered in the full-length protein. Regions of intrinsic disorder have frequently been found in DNA-binding proteins that bind multiple partners (Tantos et al. 2012). While largely disordered, they can fold on binding partners, or remain unfolded, due to slight structural preferences or charge effects. They have also sometimes been found to be involved in liquid–liquid phase separation processes. We have used carbon-detected NMR experiments in conjunction with HNN spectra to assign the isolated N-terminal extension of IncC as a first step towards examining any structural propensities in this region that may affect the function of the full-length protein.

## Methods and experiments

### Protein expression and purification

IncC NTD was expressed in *E. coli* BL21 (λDE3) cells from the plasmid pSMB315, expressing the N-terminal 105 amino acids of IncC with a 23 amino acid His-Tag from a modified pET28a vector (Batt et al. 2009). Bacteria were grown at 37 ºC in minimal M9 medium containing 1 g/L ^15^N-NH_4_Cl, 2 g/L ^13^C_6_- labelled glucose and 50 µg/mL kanamycin, and induced with 1 mM IPTG at mid log phase for 4 h before harvesting. The cells were lysed by sonication and centrifuged to remove cellular debris and ribosomes. The supernatant was purified using a Ni NTA column, in 20 mM Tris HCl buffer pH 7.5 containing 300 mM NaCl, eluting with an imidazole gradient. This was followed by size exclusion chromatography on Superdex 75, in 20 mM Tris HCl, buffer pH 7.5, 150 mM NaCl and 0.1 mM EDTA. The protein was concentrated by ultrafiltration with a 3 kDa cutoff membrane, and dialysed into 10 mM sodium phosphate buffer, pH 6.5, containing 150 mM NaCl and 0.1 mM EDTA, for NMR experiments.

### NMR spectroscopy

Spectra were obtained with 300 μM double labelled protein in 10% D_2_O, 10 mM Sodium Phosphate, 150 mM NaCl, 0.1 mM EDTA, pH 6.5, at 298 K. Carbon-detected triple resonance experiments, CON (Bermel et al. 2006); (H)CANCO, (H)CBCACON and (H)CBCANCO (Bermel et al. 2009, 2006) were used for IncC NTD backbone assignments, using a 600 MHz, Bruker spectrometer with a carbon-optimised TXO CryoProbe. Proton-detected experiments HNCO (Bermel et al. 2005; Kay et al. 1994), HNN and HNCN (Panchal et al. 2001) were used to complete and to confirm the assignments, using a 900 MHz Bruker spectrometer and a TCI probe. HSQC spectra were collected before each 3D experiment to monitor any change in signals due to protein instability, Data were processed using MddNMR (Orekhov and Jaravine 2011) and NMRpipe (Delaglio et al. 1995) software and analysed using CcpNmr Analysis ver. 2.4.2 (Vranken et al. 2005).

## Extent of assignments and data deposition

IncC NTD contains more than 70% small and charged amino acids, which are found in abundance in intrinsically disordered proteins (Uversky 2013) and considered to promote disorder. Of the 105 amino acids in this region 22 are Glycine, 12 are Alanine, and 13 are Arginine. There is only one Isoleucine and 2 Leucine residues that would normally form a hydrophobic core in a folded protein.

**Fig. 1:**
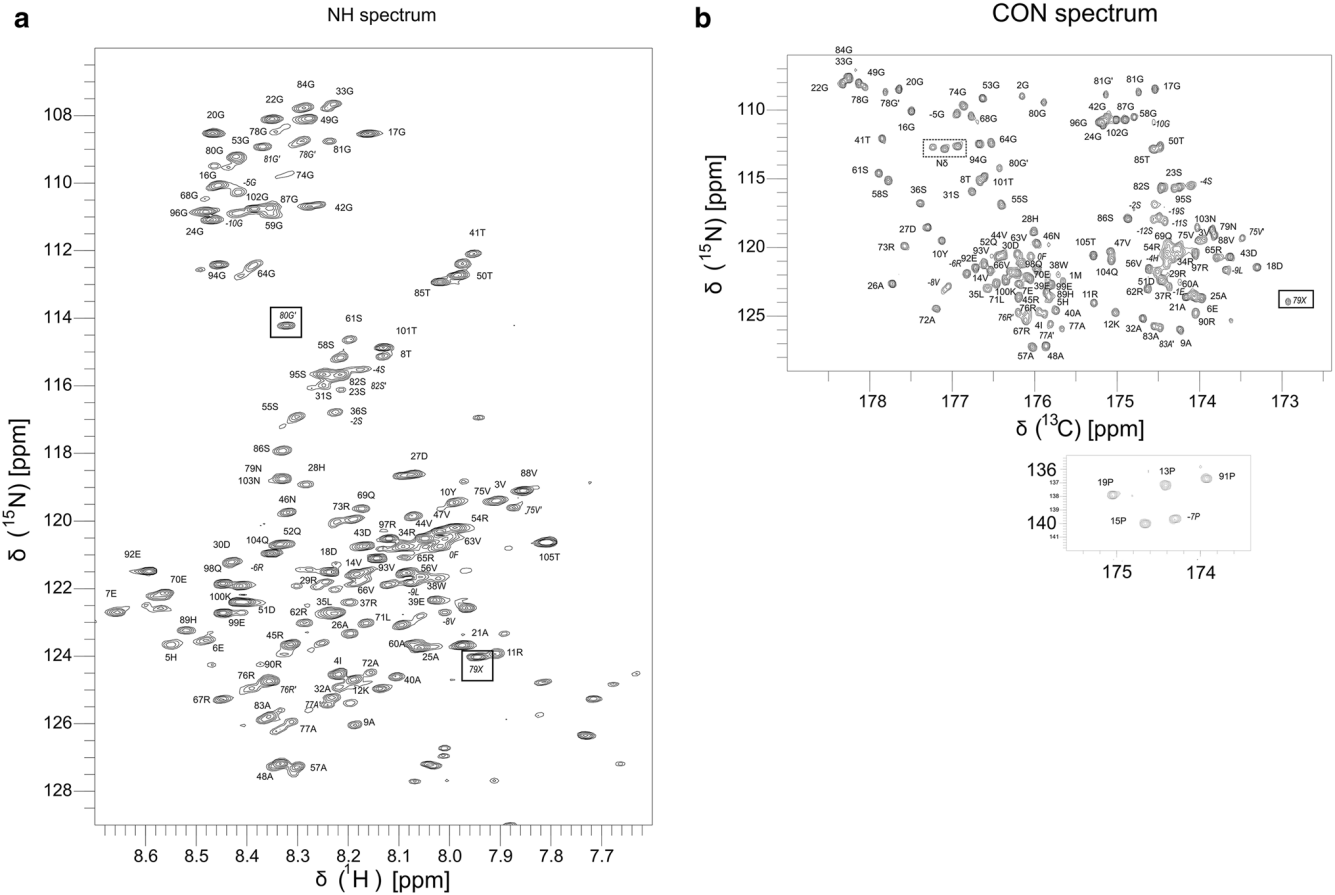
NMR Spectra of IncC NTD in 10% D_2_O, 10 mM Sodium Phosphate, 150 mM NaCl, 0.1 mM EDTA, pH 6.5, at 298 K. **a** ^1^H–^15^N HSQC spectrum, taken at 900 MHz, with NH assignments labelled with residue number and amino acid type. Peaks from the amide side chain resonances and tryptophan side chain resonance are not shown. Peaks from the N-terminal tag are labelled with negative numbers and in italics. Peaks from residues 74–83 of the deamidated species, with IsoAsp (X) are labelled with prime (′), italics and narrow font. The peaks from 79X and 80G′ are boxed. Unassigned peaks at the lower right hand side of the spectrum are thought to be from peptide degradation products. **b** CON spectrum taken at 600 MHz, labelled with the residue number and amino acid type of the N resonance. Peaks from the N-terminal tag are labelled with negative numbers and in italics. Peaks from residues 74–83 of the deamidated species, with IsoAsp (X) are labelled with prime (′), italics and narrow font. The peaks from 79X and 80G′ are boxed. Peaks from the Asn side chains are boxed with dotted lines and labelled Nδ; peaks from the Gln side chains are not shown. The proline imide N resonates at below 135 ppm and the CON peaks from these are shown in a separate box

In the ^1^H–^15^N HSQC of IncC NTD, the peaks are crowded in a narrow ~ 1 ppm (7.7–8.7 ppm) region of the ^1^H dimension and many peaks overlap (Fig. 1a). This shows that the protein is likely to be intrinsically disordered, as expected from its sequence. To overcome the overlap in the ^1^H dimension, and to assign the backbone, C-detected spectra were used; based on the CON experiment (Bermel et al. 2006) (Fig. 1b). Information from the (H)CANCO, (H)CBCANCO and (H)CBCACON spectra (Bermel et al. 2009, 2006) allowed adjacent amino acid pairs to be identified easily from the carbon shifts, but there are several duplicate pairs of amino acid in the sequence and little difference in backbone shifts for a given amino acid type. To complete the sequential assignments, and to obtain H_N_ assignments, we used HNN and HNCN experiments to connect the ^15^N shifts of three adjacent amino acids (Panchal et al. 2001), with an HNCO experiment (Kay et al. 1994) to link the C’-based assignments to the HN-based ones. With this strategy we obtained consistent backbone assignments for all the residues in IncC NTD, apart from Met 1 which does not have a peak in HNCO, and assignments for many of the residues in the N-terminal extension from vector, apart from a glycine residue and the series of His residues, that are probably overlapped. These assignments have been deposited in the BioMagResBank with ID 50740.

**Fig. 2:**
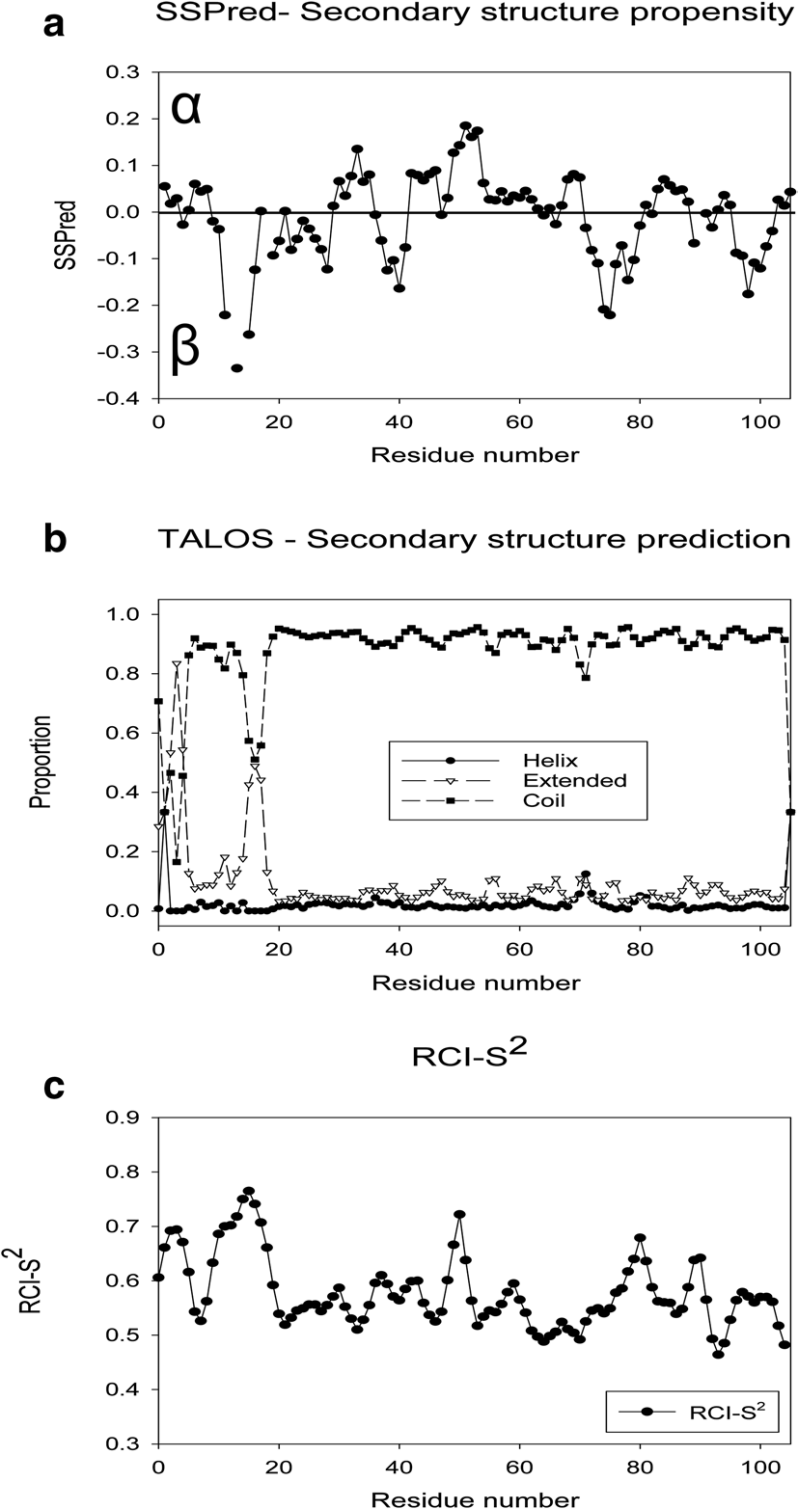
Secondary structure propensity and RCI-S^2^ order prediction from the backbone chemical shifts. **a** Secondary structure propensity of IncC NTD calculated by SSP (Marsh et al. 2006) from the Cα and Cβ shifts, with a reference offset of 0.106 ppm. Positive values indicate helical propensity while negative values indicate beta strand propensity. **b** Secondary structure of IncC NTD predicted by TALOS-N (Shen and Bax 2013) from the backbone chemical shifts. Black circles, helical propensity; white triangles, beta strand propensity; black squares, loop propensity. **c** RCI-S^2^ order number for the NH groups of IncC NTD predicted by TALOS-N based on the RCI method (Berjanskii and Wishart 2008) from the backbone chemical shifts

The secondary chemical shifts of unmodified IncC NTD were examined using several programmes, namely CSI 3.0 (Hafsa et al. 2015), DANGLE (Cheung et al. 2010), TALOS-N (Shen and Bax 2013), and SSP (Marsh et al. 2006). In each case, the analysis suggests that the protein is nearly entirely random coil. DANGLE suggested some alpha helix propensity at residues 30 and 31, while, instead, both SSP and TALOS predict some beta strand propensity around residue 15, sequence 12-KPVPGGDPG-20, although the exact residues predicted with this propensity differ in the two programmes (Fig. 2a, b). SSP gives another region of greater than 20% beta strand propensity at residues 74–75; however overall it gives only 3.1% alpha structure and 3.9% beta structure. Calculations of the NH order parameter using the RCI method (Berjanskii and Wishart 2008) in TALOS-N, suggest that the protein is dynamic with only short stretches of amino acids, namely residues 1–5, 9–18, 46–51, 78–81, 89 and 90, having order parameters above 0.6 (Fig. 2c). Only residues 11–16, at the PVP sequence with beta strand propensity, are predicted to have order parameters greater than 0.7.

A few residues were observed to have two sets of peaks in the spectra, usually with very close chemical shifts, so unlikely to be in exchange. From the HNN spectra, most of these peaks come from a series of adjacent amino acids, at residues 74–83. For the Asn79-Gly 80 pair in the set of extra peaks, the (H)CaNCO spectrum shows the Cβ of residue 79, rather than the Cα, and the HNN spectra of Gly 77 shows no connectivity to residue 79. From this we conclude that these additional peaks come from IncC NTD where Asn79 has been deamidated to give iso-Asp, with the beta carboxyl linked to Gly80. Deamidation of Asn residues occurs spontaneously in solution at neutral pH, particularly at Asn-Gly sequences where there is no steric hindrance, and in phosphate buffer (Geiger and Clarke 1987). The deamidation reaction goes via a succinimide intermediate and predominantly gives iso-Asp rather than Asp (3:1), and can also cause L- to D-isomerisation (Meinwald et al. 1986). In IncC NTD, the sequence around this residue is 78 Gly-Asn-Gly-Gly 81, so it will be highly prone to deamidation. It has been shown that deamidation in vivo is a signal for protein degradation in cytochrome c, and it has been proposed to act as a timer for other processes (Robinson and Robinson 2001), thus this modification may be functional in vivo.

Comparison of the chemical shifts of the residues in the modified and the unmodified peptide (Table 1) show that, while the largest differences in chemical shifts are, not surprisingly, at residues 79 and 80 and the differences in N shifts decrease either side of that, differences in C shifts vary across the range of residues. We were unable to assign a CON peak, or CCN peaks for the Gly 81/Ser 82 pair in the isoAsp peptide but did observe peaks corresponding to the ones expected in the nitrogen-based spectra. The carbon peaks of this pair most likely overlap with the pair in the unmodified peptide. Formation of an isoAsp at 79 changes both the backbone and the charge of the region. The effects on Arg 76, and Ser 82 may be due to the new carboxyl group forming a charge-charge interaction, or a hydrogen-bond to these residues, respectively.

**Table 1:** Difference in chemical shift of amino acid resonances around IsoAsp79 and Asn 79

Residue no	Amino acid	ΔCα (ppm)	ΔCβ (ppm)	ΔC’ (ppm)	ΔN (ppm)	ΔH_N_ (ppm)
74	G	0.06		0.53	− 0.01	− 0.01
75	V	− 0.09	− 0.06	− 0.09	0.08	0.05
76	R	− 0.10	0.08	− 0.14	0.14	0.03
77	A	− 0.03	− 0.06	0.26	0.32	0.04
78	G	0.03		0.01	− 0.27	0.05
79	X or N	− 1.55	− 1.93	− 0.54	− 5.27	0.38
80	G	− 0.16		− 0.40	− 4.77	0.14
81	G	0.06		n.d	− 0.14	− 0.13
82	S	0.13	− 0.01	0.06	0.04	0.01
83	A	0.03	− 0.31	n.d	0.02	0.09

IncC NTD spectra were taken in 10% D_2_O, 10 mM Sodium Phosphate, 150 mM NaCl, 0.1 mM EDTA, pH 6.5, at 298 K. Differences are positive if the IsoAsp species has a larger chemical shift than the normal protein

*n.d.* not determined

A few peaks were observed in the CON spectrum and HNN spectra that have yet to be identified. In particular, there are 3 C′ shifts between 169 and 171 ppm that appear to belong to a Gly/Val, a Gly/Ser and a Ser/Ser pair, respectively. These C’ shifts, and those of the Gly Cα in these pairs at ~ 44.5 ppm are similar to those of a glycine next to a succinimide (Grassi et al. 2017), but the expected sequence after the succinimide is Gly 80/Gly 81, so their identity is unclear. There may be additional chemical modifications of the peptide.

The backbone shifts of the unmodified peptide have been deposited in the BMRB data base with ID 50740. These shifts extend the data for intrinsically disordered proteins and lay the basis for NMR studies of protein and DNA interactions with IncC NTD.

## Data Availability

The assignments of the protein have been deposited in the BioMagResBank, ID 50740.
